# Surgical training in the Caribbean: The past, the present, and the future

**DOI:** 10.3389/fsurg.2023.1203490

**Published:** 2023-06-16

**Authors:** Mark S. Newnham, Dilip Dan, Ravi Maharaj, Joseph Martin Plummer

**Affiliations:** ^1^ The University of the West Indies, Mona, Kingston, Jamaica; ^2^ The University of the West Indies St. Augustine, St. Augustine, Trinidad and Tobago

**Keywords:** surgical training, collaboration, assessment, open surgery, laparoscopy

## Abstract

The six million inhabitants of these diverse English-speaking Caribbean countries are grateful to the University of the West Indies, which has been central in the independent training of surgical specialists in all areas of surgery for the past 50 years. Similar to the per capita income, the quality of surgical care, albeit acceptable, is quite variable throughout the region. Globalization and access to information have revealed that the quality of training and surgical care being delivered can be further improved. Technological advances will perhaps never be on par with higher-income countries, but collaborative ventures with global health partners and institutions can ensure that the people of the region will have appropriately trained surgical doctors and, therefore, the provision of accessible quality care will remain a staple, with even the possibility of income generation. This study reviews the journey of our structured surgical training program delivered in the region and outlines our growth plans.

## The past

The University of the West Indies (UWI), having been initially established as the University College of the West Indies under the aegis of the University of London in 1948, received its charter as a full university in 1962. Its main initial function was to provide medical practitioners to serve the needs of the Caribbean. Prior to its inception, bright young talents from the Caribbean were sent on scholarships for their medical training, mostly in the UK, with a few receiving this training in North America, to return. Others were recruited to serve in the Caribbean from these developed countries or India. Patients requiring specialist surgical care relied on a small cadre of specialist surgeons who also received their training in the UK or North America. This model of providing specialists for the region proved challenging to maintain because those trained in the USA found it financially more rewarding to stay there, while those who were successful in the Fellowship of the Royal College of Surgeons (FRCS) examinations were not always trained for consultant appointments. Additionally, those actually receiving specialist training often got trapped in the UK with the advantages of first-world living, and few made a permanent return trip to the underserved Caribbean population (1).

The stage was therefore set, and not surprisingly, the Caribbean governments urged the UWI to develop specialist training in all of the major specialties of medicine to the level of consultant. In 1967, the postgraduate doctor of medicine (DM) programs were conceptualized and commenced at the Mona Campus, with the first graduate in child health in 1973 followed by internal medicine and psychiatry in 1974.

This year (2022) marks the 50th anniversary of the start of postgraduate surgical training in the Caribbean. It had its beginnings in 1967 with the WHO/Pan American Health Organization (PAHO) Commission discussions, and subsequently, the first group of residents was admitted for general surgical training in 1972 (1). In the following year (1973), an otorhinolaryngology training program was implemented. Orthopedic surgery was soon added. The 1980s saw slow growth with high attrition rates, but with the advent of the 1990s, changes in the program directorship and challenges induced by the UK joining the European Union saw an increased interest in the program. The subspecialties of urology, cardiothoracic surgery, neurosurgery, and pediatric surgery were added, and soon afterward, there was the expansion of formal surgical training across the various campuses of the UWI with the introduction of various DMs in Trinidad and Tobago, Barbados, and The Bahamas. The turn of the millennium also saw the addition of ophthalmology (2012) and plastic surgery (2016) as the two most recent addition to our residency program. Today eight subspecialist areas provide training in addition to general surgery (Tables 1, 2).

**Table 1:** Surgery programs at the University of the West Indies.

Surgery	
General	1972 (as masters)
	1981 DM
Urology	1993
Cardiothoracic	1993
Neurosurgery	1991
Pediatric surgery	1998
ORL	1974
Orthopedics	1984
Emergency medicine	1997
Ophthalmology	2012
Plastic surgery	2016

**Table 2:** General surgery graduates from the UWI by campus.

Campus	Number of graduates
Bahamas	2
Cave Hill	16
Mona	46
Saint Augustine	20

The next step is the planned introduction of various fellowship programs such as in thoracic surgery, surgical endoscopy, and colorectal surgery.

The first three decades of its existence saw the various DM programs being present only at the Mona Campus of the UWI, with the majority of training taking place at the University Hospital of the West Indies, a few other tertiary hospitals that were accredited for training by the UWI governance body, and the Medical Faculty's Specialty Board in Surgery. This board is usually chaired by the department's head or a senior faculty and reports through the dean to the university's Board of Graduate Studies and Research. For various reasons outlined above, including the success of and wider acceptance of the program, the period of the 2000s saw the various campuses adopting and introducing postgraduate training in surgery disciplines, with general surgery commencing at the Saint Augustine Campus in 2005, Cave Hill Campus in 2012, and School of Clinical Medicine and Research, The Bahamas, in 2014.

Over the past 50 years, the various DM surgical programs from Mona had produced 46 general surgeons; 12 ophthalmologists; 20 neurosurgeons; 12 ear, nose, and throat (ENT) surgeons; seven cardiothoracic surgeons; 20 urologists; 30 orthopedic surgeons; and 12 pediatric surgeons, and our most recent graduates are three plastic surgeons. Our graduates offer consultant care at the UHWI and all the public hospitals in Jamaica and throughout the Caribbean as far south as Guyana and north as The Bahamas. In addition, they can currently be found on fellowships in the UK, Australia, and Canada, while a few occupy staff positions in the UK and North America.

The DM in general surgery at the Saint Augustine Campus was adapted from the program out of the Mona Campus with an identical structure and identical university examination process. It is accepted as a specialty degree that qualifies the successful candidate to be conferred on the Specialist Register of the Medical Board of Trinidad and Tobago and allows independent practice in the field of general surgery throughout the Caribbean. At Mona, postgraduate specialty training was traditionally sought in the UK and the USA, but these spaces have become quite competitive in recent times, hence the need to introduce postgraduate training in surgery at the Saint Augustine Campus. The environment in 2005 was indeed ripe at the time with the leadership of the UWI, and the recognized need was in sync with the focused goal of introducing this program. The program initially commenced with six candidates in 2005 at San Fernando General Hospital. It was expanded to Eric Williams Medical Sciences Complex, Mount Hope, in 2012 and the Port of Spain General Hospital. At each teaching site, a senior UWI surgery faculty facilitated the implementation and took charge of the leadership.

Since its inception in 2005, 87 candidates have entered the DM surgery program, 32 of whom (37%) were females. Of these 87 candidates, 20 (23%) have successfully exited the program and are now specialists capable of independent practice within the health service. Of these 20 specialist general surgeons, nine have completed fellowships abroad. Fellowships were in the areas of vascular, hepatopancreatobiliary, breast, advanced laparoscopy, and interventional endoscopy. Of these graduates, seven are consultants, two of whom are academic staff of the University of the West Indies. There are currently 19 active candidates in the DM General Surgery Program at the Saint Augustine Campus dispersed throughout the 5-year program, with 13 in the Part 2 phase and the remaining six in the Part 1 phase. Of these current 19 candidates, 12 (63%) are female. This is in marked distinction to the 37% females on entry from program inception and the contrast of 8% females in the first half of the program’s existence to 48% in the second half of the program’s existence. Of the 11 candidates who voluntarily withdrew from the program before the Part 1 examinations, only two were males.

The Cave Hill Campus has graduated 16 candidates from the DM General Surgery Program thus far. Of these 16, one completed a fellowship in breast surgery, one in vascular surgery, one in hepatopancreatobiliary surgery, one in advanced laparoscopy, and two in renal transplant.

The School of Medicine and Clinical Research at The Bahamas commenced postgraduate training in general surgery in 2014, and free exchange is encouraged with the Mona Campus, especially in the basic sciences at the pre-Part 1 level. To date, they have graduated three general surgeons including one who has gone on to complete a fellowship renal transplant.

## The present structure of the program

Today, the various DM surgery programs on all campuses offer a robust curriculum on par with those of North America or Europe with competency-based clinical training supplemented by didactic evidence-based tutorial sessions and certified skills-based workshops conducted in a simulated environment with the occasional wet labs. Our residents are encouraged to teach medical students and also participate in clinical research with many graduates having published in recognized regional and international journals.

The structure of the program needed a few changes over the last 50 years. It is a competitive program, and candidates are selected after a careful interview with considerations including their grade point average (GPA), clinical interest, research, and publications and references. Successful candidates then spend the first 2 years acquiring a solid foundation in the basic sciences of anatomy, physiology, pathology, and general surgery, by means of 3-monthly rotations in the disciplines of general surgery (9 months), orthopedics, neurosurgery, cardiothoracic surgery, urology, and pediatric surgery. During this period, the residents receive interactive lectures and tutorials in the basic sciences and research methods. All residents are encouraged to do the ATLS course prior to completing Part 1 of the program at the end of the first 2 years. In addition, there are mandatory workshops such as the recently introduced Laparoscopic Surgery Skills for Surgeons (LSSS) modeled after the SAGES course and others that led to the introduction of laparoscopic surgery throughout the Caribbean and a sustainable Laparoscopic Basic Skills Course as facilitated by the Caribbean College of Surgeons and the Royal College Surgeons of England (RCSEng) (2, 3). The Saint Augustine Campus of the UWI is an approved site for the RCSEng Intercollegiate Basic Surgical Skills course. At the Mona Campus, most of the skills workshops are delivered in the CHASE Carnegie Surgical Skills Laboratory based at the Department of Surgery. The CHASE Carnegie Skills Laboratory allows our residents to practice various surgical techniques including microsurgery in a simulated environment and is made accessible during their personal time. This was necessary as it was generally acknowledged by both residents and faculty as the need to increase training opportunities in minimally invasive surgery (4, 5).

Our residents are expected to be proctors in gross anatomy to our medical students and informal teaching to the medical students are encouraged. The first 2 years culminate with written and oral examinations in the basic sciences of anatomy, physiology, pathology, and principles of surgery (Part 1 examinations). The successful candidates then continue their training to spend another 3, 4, or 5 years depending on their chosen area of specialization. All residents undergo an assessment process at regular intervals to assess their progress in professionalism, communication, patient care, knowledge, and scholarly activity to identify strengths and weaknesses. Candidates are mentored during their training and are “signed off” by their supervisors as being technically competent, having high ethical standards, and exercising good leadership and judgment prior to being allowed to do their exit Part 2 examinations. Their expectations and responsibilities are increased in the apprenticeship manner much reminiscent of traditional surgical training. The dependence on and technological advances in surgery over the more recent decades have not been met by a proportionate increase in budgetary support (6) resulting that the more recent additions of neurosurgery, urology, cardiothoracic surgery and ophthalmology, and plastic surgery intrinsically have a mandatory elective period for the residents to experience surgery in a high volume first world site to enhance their training. These residents usually leave for their elective with excellent clinical acumen and work ethics and are usually good ambassadors to surgical training in the region. This elective has served these programs well with the added benefits of research collaboration and networking for fellowship training for academic surgeons.

The candidate must also participate in research activities. This now takes the form of case reports on 10 surgical cases and a clinical research project, with earlier candidates producing a “Book of 20 Case Reports.” This book must be reviewed and certified acceptable by three independent examiners before the candidate is allowed to proceed to the final DM exam. The DM Part 2 final examination is a combination of written essay-type questions as well as a grueling 90 min oral exam. Examinations are conducted twice per year by a board of regional examiners, and these are headed by a university examiner and an invited external examiner, usually a full professor of surgery from North America or Europe. The rigor of our exit examination has been praised by all external examiners as being fair and robust, with candidates being able to demonstrate their knowledge and capabilities, on par with their own training programs. The external examiners' reports often provide good feedback to the university faculty and are used for validation of the various programs. This is in addition to feedback from external faculty when our graduates are reaccepted for fellowship or are employed as consultant staff throughout the region. While this feedback is into quality improvement, a formal system of quality assurance and feedback from trainers and trainees is lacking.

## The future of surgery and surgical training in the Caribbean

The Caribbean surgeon prior to the start of our own residency programs generally would have done undergraduate medical education at the UWI and then to the USA or more commonly the UK to train in surgery and then return to practice. This still happens in a limited manner, but by far the majority of surgeons who remain in the West Indies are trained in the Caribbean (some with a fellowship training of 1–2 years externally during or after residency). The advantage of the external experience is tremendous and extremely important for the establishment of new procedures and standards (7), even if the differences in resources in being trained in a developed world university and working in a resource-restricted environment provided by most Caribbean hospitals can be challenging. Notwithstanding this fact, the future of surgery in the Caribbean is bright. The success of the DM program has resulted in adequate numbers of surgeons distributed throughout the region, even though other countries were affected by inequality of the rural–urban distribution. The prospects for surgery in the region are demonstrated by the number of UWI DM graduates who have gone on to do fellowships in the subdivision of general surgery and have returned to work in and train the next generation of Caribbean surgeons. This is facilitating a gradual move away from a multispecialist surgeon to a superspecialist surgeon. At most of our teaching hospitals, the colorectal surgeon now performs procedures in the treatment of rectal cancer, and our hepatopancreaticobiliary team performs the Whipples procedure or major hepatic resections. While most of our graduates are equipped to perform everyday laparoscopic resections, such as for cholecystitis, appendicitis, and right hemicolectomy, our more advanced procedures such as bariatric surgery are confined to a few surgical teams distributed across the region.

The overall landscape of surgical care and therefore the need for more training are not without its challenges. The Caribbean exposure provides a wide range of pathology and late presentations. The patients present with more advanced diseases as programmatic screening programs are not present. Surgical exposure is quite good for residency training, especially for common conditions and trauma. The areas for strengthening include where the pathologies are less common and where highly specialized equipment is required. With lower volumes in these areas, the exposure for the residents may be lacking. Hence, the value of fellowships for a more concentrated exposure to these areas.

A further challenge is that the resident may attain the skills (for instance, robotics) and then return to the Caribbean where they are unable to practice these skills. There are limited intergovernmental arrangements for Caribbean patients from one island to get surgery on another island where the skillset and equipment may exist. This affects patient numbers, and a highly trained subspecialty surgeon may not be fully supported, which may lead to the surgeon seeking greener pastures in the first world. Another challenge rests with the significant costs to perform high-end surgeries in the private sector.

The working hour restriction which limits US residency programs as a result of the Bell Commission (8) is not much of an issue in the Caribbean, at least for the moment. The COVID-19 pandemic has brought to the fore the importance of working conditions and the mental health of our residents. This pandemic has further reduced the size of the globe with the online format of teaching, conferences, and other means of professional development made much more accessible. We are now benefiting from training with the use of electronic and augmented learning, simulation labs with online proctoring, and soon the introduction of virtual reality in our surgical training. Nevertheless, training and procedures requiring high capital demands, such as robotics, greater accessibility of minimally invasive surgery to all members of the population, and newer techniques such as endovascular repair of aneurysms, remain a challenge. Their solutions are being explored including the introduction of specialized centers providing high-quality surgical care with the possibilities of partnerships with leading centers, thus introducing exchange and external training with capacity building. These can be mutually beneficial as leading first-world universities explore the practice of better corporate social responsibility in this era of global surgery. Currently, minimal access surgery is not accessible to all patients within some countries and even in some entire countries in the Caribbean. As we expand and saturate the markets of the countries where residency training programs exist, it is expected that there will be an overflow of trained surgeons to the islands where this skill set is required.

So what might the true future of surgery and surgical training be like in the Caribbean? Or a better question, what would we like to see as the future of surgical training in the Caribbean? Over the next few years, the number of general surgeons will gradually be reduced, and we will have superspecialists for all areas. This growth rate will be a bit slower than that in the USA, but there will always be a role for the general surgeon, especially in rural areas. It is hoped that the many “toys” that exist in the first world today will also be available in the Caribbean. However, there must be a balance between conventional traditional approaches and highly specialized approaches such as robotics. Our feeling is that this balance will be preserved based on the costs associated with these at least for the next few decades. Better yet, the Caribbean exposure may be of interest to the first world with regard to the potential for rotations for residents who may not be used to seeing traditional open surgery.

The hope is that specialized referral centers will be established and there would be government arrangements in place to manage difficult cases in all islands making healthcare accessible to all. This will also address another challenge in the Caribbean with regard to residents in the four territories of the University of the West Indies not having ready exchange rotations among the islands. CARICOM Single Market and Economy is a first step in this direction, but financial arrangements will have to be put in place for these to happen. The resident rotations which do happen in an *ad hoc* manner may be an easier challenge to address.

In terms of training, it is expected that simulation labs will be commonplace and become a requirement for the acquisition of skills to allow for a smoother transition to the operating room. Regarding electronic learning, augmented learning, and virtual reality, the bottom line is again financial, but if the UWI is to stay in the game of training surgeons, this is a key investment. The leadership of the UWI in partnership with the various national or regional surgical bodies, including the Caribbean College of Surgeons, must see this as an imperative (9). There are various “low-hanging fruits,” such as greater cooperation at the postgraduate level, and not just examinations but, in fact, teaching and training. Didactic teaching sessions should be held simultaneously across the various teaching sites reducing costs and taking advantage of expertise. The COVID-19 pandemic has taught us that there are so many opportunities to use the Internet for education and training. Online proctoring has already been used in the Caribbean for assisting surgeons and will become more commonplace. This will also be the same for the surgical training of residents. Well-crafted training grants including partnerships with sister faculties from North America will provide training opportunities, and by building centers of excellence in various areas, they will be income-generating and increase opportunities for fellowship training, while expanding medical tourism, especially with the Diaspora as the intended target.

The Caribbean has always been considered too small to generate high-powered research. This false perception must change. We do have the materials and caseload as a combined unit to do multicenter randomized controlled trials. We may not have the resources or the skillset to do all of the work, but we have a more agile workforce and policies that will facilitate the research process. This is certainly an area that needs the right attention. We would have to work out common standards for a start, and these may be a bit different for our population as compared to the USA. The National Comprehensive Cancer Network (NCCN) has recently developed guidelines for the Caribbean in cancer care while the University Hospital of the West Indies and the Association of Surgeons in Jamaica published Guidelines for Selected Surgical Diseases (10). These are areas where guidelines specific to the Caribbean were developed by consensus and are being implemented. We must look at other regionally important surgical diseases such as gastrointestinal bleeding, diverticulitis, and diabetic foot care just to name a few.

Another area that is required and to which we must strive is the creation of an independent examination board for our graduating residents. As it exists now, we have a fairly rigid process using external examiners combined with regional examiners from campuses to which the student does not belong. Nevertheless, those are the same UWI lecturers who teach, examine, and award the professional degree. While this approach was initially necessary, the UWI should now be responsible for training and an examination body for licensure. Similar to the undergraduate medical degree, our postgraduate medical training should be accredited by the Caribbean Accreditation Authority in Medicine and other health professionals. Again, the partnership with the Caribbean College of Surgeons can provide a solution. This needs the buy-in of the UWI and also CARICOM. Additionally, surgical leaders in the region must participate and take advantage of established initiatives that will benefit the region, such as the Lancet Commission on Global Surgery and the Latin American Indicator Research Collaboratory aimed at increasing access to timely, high-quality, affordable surgical care, and data-driven surgical education and training, respectively (11, 12).

In conclusion, the Caribbean is proud of the quality of surgical training being delivered currently, and the leadership at the University of the West Indies is aware of its limitations. Investments in infrastructure are needed, and surgical fellowships in specialized areas will have to be created. Better distribution of human resources is needed, and greater cooperation with government and institutions external to the region has a role. Appropriately placed income-generating specialized centers of excellence meeting the needs of the region while facilitating research with first-world institutions is the ultimate goal. Thankfully, the leadership is aware and capable as our failure to act will stagnate the future of Caribbean surgery and surgical training. The people of the region deserve and will receive better.

## Data Availability

The original contributions presented in the study are included in the article; further inquiries can be directed to the corresponding author.
